# ATTITUDES TOWARD AND PREFERENCE FOR ADVANCE CARE PLANNING AMONG OLDER KOREAN AMERICANS

**DOI:** 10.1093/geroni/igz038.1513

**Published:** 2019-11-08

**Authors:** Michin Hong, Hyunjun Noh, Kyeongmo Kim

**Affiliations:** 1 Indiana University, Indianapolis, Illinois, United States; 2 the university of alabama, Tuscaloosa, Alabama, United States; 3 Virginia Commonwealth University, Richmond, Virginia, United States

## Abstract

Advance care planning (ACP), referring to a decision-making process for end-of-life (EOL) care in advance, is critical in ensuring satisfaction with care and quality of life among patients and caregivers at the EOL. However, Korean Americans consistently report lower levels of ACP engagement compared to whites, indicating their potential vulnerability at the EOL. To gain insight into strategies to address the disparity, this qualitative study explored attitudes toward and preference for ACP among older Korean Americans. We conducted three focus group interviews with 31 older Korean Americans. Through iterative data analysis process, four themes emerged: 1) moderate levels of awareness of and prior experiences with ACP: 2) favorable attitudes toward ACP, while concerned about non-compliance by children: 3) preference for natural dying without life prolonging treatments and pain: and 4) preference for physician-initiated ACP. Our findings suggest the necessity of culturally tailored approaches to promote ACP for this population.

